# A microscopic traffic characterization considering the impact of density on carbon emissions from CAVs

**DOI:** 10.1038/s41598-026-37851-x

**Published:** 2026-02-25

**Authors:** Zawar Hussain Khan, Faryal Ali, Thomas Aaron Gulliver, Mohammad Alsaffar, Ahmed B. Altamimi

**Affiliations:** 1 Department of Computer Engineering, College of Computer Science and Engineering, University of Ha’il, Ha’il, Saudi Arabia; 2 Department of Electrical and Computer Engineering, University of Victoria, Victoria, BC V8W 2Y2 Canada; 3 Department of Information and Computer Science, College of Computer Science and Engineering, University of Ha’il, Ha’il, Saudi Arabia

**Keywords:** Density, Microscopic traffic, CAVs, Traffic emissions, ID model, Stability analysis, Statistical analysis, Engineering, Environmental sciences, Mathematics and computing

## Abstract

The continuous growth of traffic and transportation activities across the globe has led to persistent congestion and greenhouse gas emissions from vehicles. Thus, it is important to develop a traffic model to mitigate congestion and air pollution. In this paper, a microscopic traffic model characterizing traffic emissions based on density is proposed. Field experiments were performed, and the data collected were analyzed to obtain the relationship between density and $\:\mathrm{C}{\mathrm{O}}_{2}$ emissions. Then, the connected autonomous vehicle (CAV) parameter was incorporated, and a new traffic model was developed by integrating it into the intelligent driver (ID) model. The ID model characterizes traffic based on a constant exponent and ignores traffic emissions and CAV behavior. The proposed model can provide details of vehicle emissions under varying traffic densities. Further, the stability analysis illustrates that the proposed model results in more stable traffic compared to the ID model, as it is based on real-world traffic parameters. For performance analysis, the proposed and ID models are compared over a $\:1000$ m circular road using MATLAB. Results suggest that the proposed model traffic behavior is more realistic and the variations in traffic speed, density, and acceleration based on the traffic emissions are small compared to the ID model, thereby resulting in lower emissions. Further, the statistical analysis indicates a lower variability in the proposed model, reflecting lower emissions and stable traffic behavior.

## Introduction

Climate change due to energy consumption and air pollution has gained considerable importance in recent years and transportation is a major contributor^1^. $\:\mathrm{C}{\mathrm{O}}_{2}$ from burning fossil fuels is about 65% of greenhouse gas (GHG) emissions worldwide^2^ and the transportation sector contributes about $\:25$% of $\:\mathrm{C}{\mathrm{O}}_{2}$ emissions. These emissions are increasing rapidly due to the growing traffic volume worldwide. Thus, reducing GHG emissions from vehicles is one of the biggest global challenges^3^. Annual traffic emissions are expected to double by 2050^4^. There has been an $\:80$% increase in these emissions in the United States since 1980^5^, and in 2020 accounted for 45% of the air pollution^6^. In Canada, there were $\:156$ megatons of $\:\mathrm{C}{\mathrm{O}}_{2}$ emissions from road traffic in $\:2019$ which is $\:21$% of the national GHG emissions^7^. In Saudi Arabia, there were $\:3.68$ tonnes per capita of $\:\mathrm{C}{\mathrm{O}}_{2}$ emissions from the transportation sector in 2021, which is $\:22$% of the national $\:\mathrm{C}{\mathrm{O}}_{2}$ emissions^8^. In 2022, approximately $\:26.6$% of households in Ha’il suffered from air pollution^9^. According to the King Abdullah Petroleum Studies and Research Centre, $\:\mathrm{C}{\mathrm{O}}_{2}$ emissions from transportation are predicted to be around 184 million tonnes in 2030^10^.

Traffic emissions degrade air quality and adversely impact biological systems. They also contribute to health issues such as respiratory problems and heart diseases^11^. Therefore, it is crucial to characterize these emissions to enhance air quality and mitigate the associated hazards. Methods for characterizing emissions consider traffic conditions, speed, emission levels, and fuel consumption^12,13^. An average speed model to assess fuel consumption ratios was developed in^14^. However, these ratios were estimated using a specific road segment and hence cannot be used for general road sections and speeds exceeding $\:60$ km/hr^15^. The VT-micro model was proposed in^16^ to determine fuel consumption and traffic emissions considering vehicle speed and acceleration. The California Air Resources Bureau proposed the emission factors (EMFAC) model for fuel consumption and vehicle emission estimation^12^. The package emissions from road transport (COPERT)^17^ was created by the Environmental Protection Agency. However, these models have a large number of parameters, so they are complex and have limited scope^12^.

Recently, autonomous vehicle technologies have been developed to enhance safety and fuel efficiency^18^. Connected autonomous vehicles (CAVs) have been designed to improve capacity, safety, comfort, mobility, and environmental sustainability^19^, and thus there is considerable interest in CAVs for GHG reduction^20^. Particularly^21^, recently demonstrated that CAVs can improve the capacity of mixed traffic flow, further reinforcing their value in future transportation networks. It is anticipated that CAVs will define the future of transportation systems, and $\:75$% of vehicles will be CAVs by $\:2050$^19,22^.

The effect of CAVs on fuel consumption and traffic emissions has been investigated in the literature. Emissions due to connected vehicle technology (CVT) were assessed via simulation in^23^. It was determined that CVT can reduce fuel consumption and emissions in all traffic conditions. CAV emissions at intersections with signals were investigated in^24^. The field study results in^25^ show that reductions in $\:\mathrm{C}{\mathrm{O}}_{2}$ emissions from CAVs are a function of vehicle speed and are in the range $\:5-7$%. In^26^, CAVs were shown to improve traffic stability and mitigate congestion such as stop-and-go waves. This resulted in a reduction in emissions of $\:13-73$%. However, traffic conditions were not considered, and hence the results are not related to real-world environments^27^. Further^24^, introduced a trajectory optimization approach for CAVs at signalized intersections to reduce traffic congestion and emissions^28^. developed a deep reinforcement learning method, whereas^29^ presented a speed harmonization method to improve traffic control, leading to energy efficient and low emission transportation systems. These studies investigate how CAVs can substantially reduce energy consumption and emissions across various traffic scenarios, indicating that the adoption of CAVs will affect the traffic dynamics. Therefore, accurately modeling these dynamics is important to better characterize traffic emissions.

Traffic models can be microscopic or macroscopic. Microscopic models focus on individual vehicles and integrate parameters such as speed, position, time, and headway^30^. Whereas macroscopic models employ parameters including density and speed to describe the aggregate behavior of traffic^2^. There are various well-established microscopic models for conventional vehicles. However, with the introduction of automation and communication technology, the vehicle behavior may significantly change at a microscopic level^31^. Therefore, new microscopic models that include CAVs need to be developed.

Moreover, modeling the individual behavior of CAVs presents significant potential to minimize energy consumption and emissions^32^. Unlike trajectory optimization, microscopic modeling emphasizes not only the optimal path for individual vehicles but also signifies the vehicle interaction and dynamic collaboration of the entire platoon in different traffic scenarios including dense traffic. Therefore, developing a microscopic model for CAVs considering traffic emissions is essential for investigation. A microscopic model for CAVs was introduced in^32^to reduce energy consumption and emissions while incorporating variations in spacing between vehicles and the effect of forward and rearward vehicles. In^33^, a microscopic CAV model was developed based on the speed of forward vehicles to lower emissions, and in^34^, microscopic models were employed to investigate the impact of CAV on energy consumption and emissions on expressways. A model for CAVs based on speed harmonization was proposed in^29^ to reduce emissions under mixed traffic. However, studies on microscopic emission modeling for CAVs are limited, particularly in terms of fully integrated, data-driven models that explicitly couple traffic dynamics with emission modeling under real-world traffic conditions. The model presented here is developed based on roadside data and more accurately captures microscopic driving behavior relevant to emissions.

**Fig. 1:**
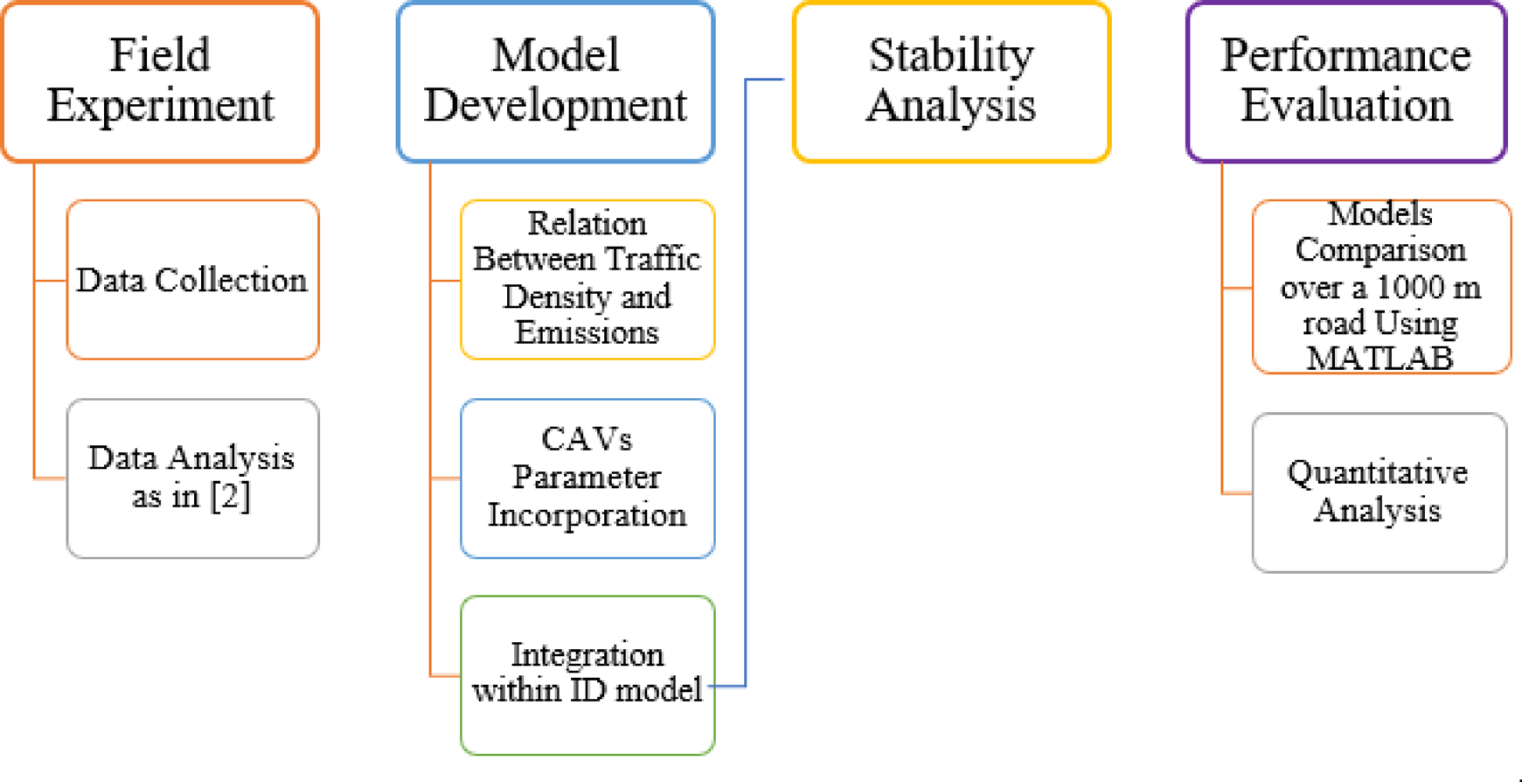
The research methodology.

In this study, a new microscopic traffic model for CAVs is proposed to realistically characterize traffic emissions based on the traffic density. First, a field experiment was performed to determine the impact of traffic density on emissions on two routes (morning and evening) spanning $\:7.7$ km and $\:7.1$ km. The morning route was from Phase 2, Hayatabad, to the University of Engineering and Technology (UET) Peshawar, and the evening was from UET Peshawar to Phase 2, Hayatabad, located in Khyber Pakhtunkhwa, Pakistan. Then, the data is analyzed as in^2^, and a new microscopic model is developed by integrating the CAV behavior and incorporating it into the intelligent driver (ID) model, with the driving behavior influencing traffic dynamics and emissions. The ID model is a state-of-the-art model and has been shown to produce better results than previous models^30^. The stability analysis of the proposed model is compared with the ID model. Both models are discretized for simulation using the Euler technique in MATLAB. The simulations are performed under identical boundary conditions and vehicle parameters. The comparison focuses on emission-related indicators such as speed variability and acceleration/deceleration characteristics. The results obtained demonstrate that the proposed model better characterizes traffic behavior based on emissions compared to ID model. The flow chart of methodology is presented in Fig. 1.

This paper is organized as follows. In Sect. 2, traffic models are presented. Section 3 provides a stability analysis, and Sect. 4 presents the performance of the proposed and ID models. A summary of the paper is given in Sect. 5.

## Traffic models

**Fig. 2:**
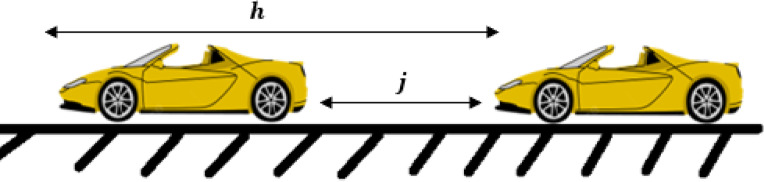
ID model parameters illustration.

In the ID model^35^, acceleration is characterized as.

1 $$\:a=A\left(1-{\left(\frac{v}{{v}_{m}}\right)}^{\delta\:}-{\left(\frac{s}{h}\right)}^{2}\right)$$

where $\:A$ is the maximum acceleration, $\:v$ is the vehicle speed, $\:{v}_{m}$ is the desired speed, $\:\delta\:$ is the acceleration exponent, $\:h$ is the distance headway, and $\:s$ is the desired distance headway given by.

2 $$\:s=j+Tv+\frac{v\varDelta\:v}{2\sqrt{AB}}$$

where $\:\varDelta\:v$ is the difference in speed, $\:T$ is the time headway, that is, the time required to align to the forward traffic condition, and the distance covered during this time is the distance headway^36^. $\:B$ is the deceleration, and $\:j$ is the jam spacing as shown in Fig. 2. The ID model employs (1) and (2) to characterize traffic behavior and indicates that traffic behavior is determined by a $\:\delta\:$ which is a constant and results in uniform driver behavior in different traffic conditions and is not related to traffic flow physics. Thus, it can result in unrealistic behavior. Furthermore, the ID model does not consider traffic emissions such as CO₂ or the behavior of CAVs, which significantly affect traffic dynamics. These are essential for realistically assessing traffic behavior and air quality on roads.

An acceleration exponent is proposed here to characterize $\:\mathrm{C}{\mathrm{O}}_{2}$ emission based on the traffic density and provide a more realistic representation of traffic behavior. Then, $\:\delta\:$ represents the sensitivity of emissions to changes in traffic density. During free flow, traffic density is low, allowing the vehicles to accelerate smoothly and maintain lower emissions. In congestion the density is high, and vehicles accelerate and decelerate more, leading to a large amount of $\:\mathrm{C}{\mathrm{O}}_{2}$ emissions released. However, the proposed model does not impose a constraint on driver behavior for an explicit emission-based optimization, rather emissions are treated as an outcome of traffic dynamics.

Field experiments were conducted by driving a vehicle for 2 trips over a road segment in Peshawar, Pakistan. The morning trip was between $\:8:39$ AM and $\:8:54$ AM, and the evening trip was between $\:5:20$ PM and $\:5:35$ PM. Pollution emissions from vehicle exhaust during trips were obtained using a dynamometer. The data was collected from the engine control unit (ECU) using an On-Board Diagnostic-II scanner connected to a BotlnckDectr^37^ app. This setup enabled real-time collection of emission data when a vehicle traversed a route. The data was transmitted to the Amazon Web Services (AWS) cloud and is then analyzed. The regression technique was utilized to obtain the relationship between $\:\mathrm{C}{\mathrm{O}}_{2}$ emissions and density as^2^.

3 $$\:{E}_{c{o}_{2}}=1.899-0.03599\rho\:+0.000157{\rho\:}^{2}$$

The change in vehicle emissions with density is.

4 $$\:\frac{{dE}_{c{o}_{2}}}{d\rho\:}=-0.03599+0.000314\rho\:$$

Traffic flow is the product of speed and density.

5 $$\:f=v.\rho\:$$

and the flow is large with a small time headway $\:T$ and vice versa^38^. Thus, (4) can be written as.

6 $$\:\frac{{dE}_{c{o}_{2}}}{d\rho\:}=-0.03599+\frac{0.000314}{Tv}$$

Further, reaction time influences traffic emissions^38^. When the reaction time is short, drivers respond quickly to changes in traffic and maintain a smoother and more consistent speed. Thus, the vehicles accelerate and decelerate more gradually, resulting in lower emissions. Conversely, when the reaction time is large, the driver reacts slowly to changes in traffic, causing delays in braking or accelerating. This often leads to stop-and-go waves in traffic, that is, the acceleration and deceleration are more frequent, resulting in higher emission levels.

Moreover, emissions are impacted by the ratio of distance headway $\:h$ to safe distance headway $\:{h}_{s}$. When $\:h\ge\:{h}_{s}$, the traffic density is low and vehicles can maintain a large distance headway, resulting in smooth acceleration and low emissions. Whereas, when $\:h<{h}_{s}$ the traffic density is high, leading to large acceleration and deceleration and consequently high emissions.

Thus, the proposed acceleration exponent δ is.

7 $$\:\delta\:=\left(-0.03599+\frac{0.000314}{Tv}\right)\left(\frac{h}{{h}_{s}}\right){r}_{c}\:\:$$

where $\:{r}_{c}$ is the CAV reaction time and is smaller than human driven vehicle reaction time. This is because of the sensors and communication technology used in CAVs, which process and respond to traffic conditions much faster than humans. Equation (7) represents $\:\delta\:$ as a reaction time and distance headway dependent relation which characterizes vehicle interaction and acceleration/deceleration behavior under different traffic conditions, rather than as an independently optimized control variable. $\:\delta\:$ captures the deviations from the desired speed as traffic density varies, while considering the impact of traffic emissions. The units of $\:\delta\:$ are (kg$\:\mathrm{C}{\mathrm{O}}_{2}$/veh)s.

Substituting (7) in place of $\:\delta\:$ in (1) gives the proposed model as.

8 $$\:a=A\left(1-{\left(\frac{v}{{v}_{m}}\right)}^{\left(-0.03599+\frac{0.000314}{Tv}\right)\left(\frac{h}{{h}_{s}}\right){r}_{c}\:\:}-{\left(\frac{s}{h}\right)}^{2}\right)$$

This model characterizes the traffic that considers traffic emissions and can predict emissions dynamically, depending on the road traffic density. Thus, it leads to a more realistic and environmentally relevant traffic behavior compared to the ID model. With the proposed model, traffic behavior varies according to the time headway and reaction time. A longer time headway allows vehicles more time to adapt to upstream traffic conditions, corresponding to lower density. This results in smoother traffic flow and reduced emissions. Conversely, shorter time headways reduce adaptation time, leading to higher density. Thus, results in more frequent acceleration and deceleration events, higher engine load, and increased emissions. Conversely, the ID model produces similar behavior for all traffic conditions. Moreover, the proposed model can account for CAVs dynamically adjusting their speed and headway to surrounding traffic conditions, leading to reduced congestion and lower emission levels, which is not achievable with the ID model.

9 $$\:{h}_{e}=\left(j+Tv\right){\left(1-{\left(\frac{v}{{v}_{m}}\right)}^{\left(-0.03599+\frac{0.000314}{Tv}\right)\left(\frac{h}{{h}_{s}}\right){r}_{c}}\right)}^{-0.5}$$

In microscopic traffic models, density is expressed as $\:\rho\:=\frac{1}{{h}_{e}}$^39^, where $\:{h}_{e}$ is the equilibrium distance headway obtained by substituting (2) in (8). At equilibrium, $\:\varDelta\:v=0$This indicates that with the proposed model, distance headway between vehicles at equilibrium is based on tCAV reaction time and time headway and is more realistic than the constant in the ID model. From (5), $\:f=v/{h}_{e}$ and substituting this in (9) gives the traffic flow for the proposed model.

, thus.

10 $$\:f=\frac{v{\left(1-{\left(\frac{v}{{v}_{m}}\right)}^{\left(-0.03599+\frac{0.000314}{Tv}\right)\left(\frac{h}{{h}_{s}}\right){r}_{c}}\right)}^{0.5}}{\left(j+Tv\right)}$$

## Stability analysis

For stability analysis, an infinite road with uniform characteristics is considered so the driver and vehicle characteristics are identical. Vehicles maintain the same (equilibrium) distance headway $\:{h}_{e}$ and speed $\:{v}_{e}$. Small variations in this distance headway are denoted by $\:a$ and changes in speed by $\:b$. Then, the distance headway during vehicle adjustments can be expressed as.

11 $$\:h={h}_{e}+a,\:$$

and the corresponding speed is.

12 $$\:v={v}_{e}+b.$$

Changes in the distance headway over time lead to variations in speed during the alignment process between the subject vehicle $\:S$ and leading vehicles $\:l$ so^40^.

13 $$\:a\left(t\right)=\frac{da}{dt}={b}_{l}-{b}_{S}.$$

The variation in speed over time is^41^.

14 $$\:b\left(t\right)=\frac{db}{dt}={P}_{h}{a}_{s}+{(P}_{v}+{P}_{\varDelta\:v}){b}_{s}-{P}_{\varDelta\:v}{b}_{l}$$

where $\:{P}_{h}$, $\:{P}_{v}$ and $\:{P}_{\varDelta\:v}$ are.

$$\:{P}_{h}=\frac{\partial\:P}{\partial\:h},\:{P}_{v}=\frac{\partial\:P}{\partial\:v}\:\mathrm{a}\mathrm{n}\mathrm{d}\:{P}_{\varDelta\:v}=\frac{\partial\:P}{\partial\:\varDelta\:v}$$

Using Fourier-Ansatz, (13) and (14) can be expressed as.

15 $$\:a\left(t\right)=\widehat{a}{e}^{\epsilon\:t+ip}$$

16 $$\:b\left(t\right)={\widehat{b}e}^{\epsilon\:t+ip}$$

or

17 $$\:\left(\begin{array}{c}a\left(t\right)\\\:b\left(t\right)\end{array}\right)=\left(\begin{array}{c}\widehat{a}\\\:\widehat{b}\end{array}\right){e}^{\epsilon\:t+ip}$$

where $\:\epsilon\:=\mu\:+i\psi\:\:$is the rate of change of traffic oscillations during adjustment, and $\:i=\sqrt{-1}$. The real part $\:\mu\:$ represents the change in amplitude, while $\:\psi\:=\frac{2\pi\:}{\tau\:}$ denotes the frequency of oscillation with period $\:\tau\:$. Further, $\:p$ is the phase shift corresponding to driver delay^41^, while $\:\widehat{a}$ and $\:\widehat{b}$ are the adjustments in distance headway and speed, respectively.

Substituting (17) in (13) and (14) gives.

18 $$\:a\left(t\right)=b-b{e}^{ip}$$

19 $$\:b\left(t\right)={P}_{s}a{e}^{ip}+\left({P}_{v}+{P}_{\varDelta\:v}\right)b{e}^{ip}-{P}_{\varDelta\:v}b$$

A model is stable if the eigenvalues $\:\epsilon\:$ of the Jacobian matrix $\:J$ have negative real components. These eigenvalues can be obtained from.

20 $$\:\left|J-\left(\begin{array}{cc}\epsilon\:&\:0\\\:0&\:\epsilon\:\end{array}\right)\right|=0$$,

where $$\:J=\left(\begin{array}{cc}{j}_{11}&\:{j}_{12}\\\:{j}_{21}&\:{j}_{22}\end{array}\right)$$ and $\:{j}_{11}$ and $\:{j}_{21}$ are the partial derivatives of (18) and (19) with respect to $\:a$ and $\:{j}_{12}$ and $\:{j}_{22}$ are the partial derivatives of (18) and (19) with respect to $\:b$. Then.

21 $$\:J={e}^{ip}\left(\begin{array}{cc}0&\:{e}^{-ip}-1\\\:{P}_{s}&\:\left({P}_{v}+{P}_{\varDelta\:v}\right)-{P}_{\varDelta\:v}{e}^{-ip}\end{array}\right),$$

and substituting this in (20) gives.

22 $$\:\left(\begin{array}{cc}\epsilon\:&\:1-{e}^{-ip}\\\:-{P}_{s}&\:\epsilon\:-{P}_{v}-{P}_{\varDelta\:v}+{P}_{\varDelta\:v}{e}^{-ip}\end{array}\right)=0$$,

so,

23 $$\:{\:\:\:\epsilon\:}^{2}+(-{P}_{v}-{P}_{\varDelta\:v}+{P}_{\varDelta\:v}{e}^{-ip})\epsilon\:+{P}_{s}\left(1-{e}^{-ip}\right)=0$$

$$\:\mathrm{L}\mathrm{e}\mathrm{t}\:G\left(p\right)=-{P}_{v}-{P}_{\varDelta\:v}+{P}_{\varDelta\:v}{e}^{-ip},\:\mathrm{a}\mathrm{n}\mathrm{d}\:H\left(p\right)={P}_{s}\left(1-{e}^{-ip}\right)\:\mathrm{s}\mathrm{o}$$

24 $$\:{\:\:\:\epsilon\:}^{2}+G\left(p\right)\epsilon\:+H\left(p\right)=0\:$$

Then the eigenvalues are.

25 $$\:{\epsilon\:}_{\mathrm{1,2}}=-\frac{G\left(p\right)}{2}\left(\:1\pm\:\sqrt{1-\frac{4H\left(p\right)}{{G}^{2}\left(p\right)}}\right)$$.

A model is string stable^41^ if the real parts of the eigenvalues are negative. Then traffic oscillations decrease over time, resulting in a stable and smooth flow^42^. Conversely, instability arises when traffic oscillations increase as in congestion, in which case the acceleration can be high^39^. In addition, $\:p\to\:0$ due to instability which decreases the delay between flow changes^41^. Approximating $\:G\left(p\right)$ and $\:H\left(p\right)$ using Taylor series for a small delay, i.e. $\:p\to\:0$, gives.

26 $$\:G\left(p\right)\:=-{P}_{v}-i{P}_{\varDelta\:v}p$$

27 $$\:H\left(p\right)\:=i{P}_{s}p+\frac{{P}_{s}}{2}{p}^{2}$$

At equilibrium, from^41^.

28 $$\:{P}_{s}=-{v}_{e}^{{\prime\:}}\left({h}_{e}\right){P}_{v},$$

where $\:{v}_{e}^{{\prime\:}}\left({h}_{e}\right)$ is the change in speed with an equilibrium gap at equilibrium. Then (27) becomes.

29 $$\:H\left(p\right)=-i{v}_{e}^{{\prime\:}}\left({h}_{e}\right){P}_{v}-\frac{{v}_{e}^{{\prime\:}}\left({h}_{e}\right)}{2}{P}_{v}.\:\:\:$$

Let.

$$\:G\left(p\right)={x}_{1}+{x}_{2}p$$

30 $$\:H\left(p\right)={y}_{1}p+{y}_{2}{p}^{2\:}$$

where,

31 $$\:{x}_{1}=-{P}_{v}{x}_{2}=-i{P}_{\varDelta\:v}{y}_{1}=-i{v}_{e}^{{\prime\:}}\left({h}_{e}\right){P}_{v}=i{v}_{e}^{{\prime\:}}\left({h}_{e}\right){x}_{1}{y}_{2}=-\frac{{v}_{e}^{{\prime\:}}\left({h}_{e}\right)}{2}{P}_{v}=\frac{{v}_{e}^{{\prime\:}}\left({h}_{e}\right)}{2}{x}_{1}$$

Approximating the square root in (25) using Taylor series gives.

32 $$\:\sqrt{1-\frac{4H\left(p\right)}{{G}^{2}\left(p\right)}}=1-\frac{2H\left(p\right)}{{G}^{2}\left(p\right)}-\frac{2{H}^{2}\left(p\right)}{{G}^{4}\left(p\right)},$$

which results in,

33 $$\:{\epsilon\:}_{2}=\frac{-H\left(p\right){G}^{2}\left(p\right)-{H}^{2}\left(p\right)}{{G}^{3}\left(p\right)}$$

From (30).

34 $$\:{\epsilon\:}_{2}=-\frac{{y}_{1}}{{x}_{1}}p+\left(\frac{{y}_{1}{x}_{2}}{{x}_{1}^{2}}-\frac{{y}_{2}}{{x}_{1}}-\frac{{y}_{1}^{2}}{{x}_{1}^{3}}\right){p}^{2}$$,

and substituting (31) gives.

35 $$\:{\epsilon\:}_{2}=-i{v}_{e}^{{\prime\:}}\left({h}_{e}\right)p+\frac{{v}_{e}^{{\prime\:}}\left({h}_{e}\right)}{{P}_{v}}\left[-\frac{2{P}_{\varDelta\:v}-{P}_{v}}{2}-{v}_{e}^{{\prime\:}}\left({h}_{e}\right)\right]{p}^{2}.$$

The real part of (35) is the growth rate, i.e. the rate at which the amplitude of the traffic oscillations changes. The model is stable if this is negative since.

36 $$\:{v}_{e}^{{\prime\:}}\left({h}_{e}\right)\:\ge\:0\:\mathrm{a}\mathrm{n}\mathrm{d}\:{P}_{v}<0.$$

Then from^43^, the string stability criteria is $\:-\frac{2{P}_{\varDelta\:v}-{P}_{v}}{2}-{v}_{e}^{{\prime\:}}\left({h}_{e}\right)$, or.

37 $$\:{v}_{e}^{{\prime\:}}\left({h}_{e}\right)\le\:-\frac{{P}_{v}}{2}-{P}_{\varDelta\:v}$$

From (36) and (37), the product of $\:-\frac{2{P}_{\varDelta\:v}-{P}_{v}}{2}-{v}_{e}^{{\prime\:}}\left({h}_{e}\right)$ and $\:\frac{{v}_{e}^{{\prime\:}}\left({h}_{e}\right)}{{P}_{v}}$ indicates that the real part of $\:{\epsilon\:}_{2}$ is negative.

At equilibrium, $\:{P}_{v}$ and $\:{P}_{\varDelta\:v}$ for the proposed model given by (8) are.

$$\:{P}_{v}=A\left(-\frac{\left(-0.03599+\frac{0.000314}{Tv}\right)\left(\frac{h}{{h}_{s}}\right){r}_{c}{{v}_{e}\left({h}_{e}\right)}^{\left(\left(-0.03599+\frac{0.000314}{Tv}\right)\left(\frac{h}{{h}_{s}}\right){r}_{c}\right)-1}}{{v}_{m}^{\left(-0.03599+\frac{0.000314}{Tv}\right)\left(\frac{h}{{h}_{s}}\right){r}_{c}}}-\frac{2T\left(j+{v}_{e}\left({h}_{e}\right)T\right)}{{h}_{e}^{2}}\right)$$

and

$$\:{P}_{\varDelta\:v}=-\frac{{v}_{e}\left({h}_{e}\right)}{{h}_{e}}\sqrt{\frac{A}{B}}\left(\frac{j+{v}_{e}\left({h}_{e}\right)T}{{h}_{e}}\right)$$

Substituting $\:{P}_{v}$ and $\:{P}_{\varDelta\:v}$, the string stability criteria for the proposed model from (37) is.

38 $$\begin{gathered} (v_{e}^{\prime }\left( {{h_e}} \right) \leqslant \frac{{A\left( {\begin{array}{*{20}{c}} {\left( { - 0.03599+\frac{{0.000314}}{{Tv}}} \right)\left( {\frac{h}{{{h_s}}}} \right){r_c}h_{e}^{2}{v_e}{{\left( {{h_e}} \right)}^{\left( {\left( { - 0.03599+\frac{{0.000314}}{{Tv}}} \right)\left( {\frac{h}{{{h_s}}}} \right){r_c}} \right) - 1}}+2Tjv_{m}^{{\left( { - 0.03599+\frac{{0.000314}}{{Tv}}} \right)\left( {\frac{h}{{{h_s}}}} \right){r_c}}}+} \\ {2{v_e}\left( {{h_e}} \right){T^2}v_{m}^{{\left( { - 0.03599+\frac{{0.000314}}{{Tv}}} \right)\left( {\frac{h}{{{h_s}}}} \right){r_c}}}} \end{array}} \right)}}{{2{{\left( {{h_e}} \right)}^2}v_{m}^{{\left( { - 0.03599+\frac{{0.000314}}{{Tv}}} \right)\left( {\frac{h}{{{h_s}}}} \right){r_c}}}}} \hfill \\ +\frac{{{v_e}\left( {{h_e}} \right)\sqrt {AB} \left( {{h_e}+T{v_e}\left( {{h_e}} \right)} \right)}}{{{{\left( {{h_e}} \right)}^2}B}} \hfill \\ \end{gathered}$$

This indicates that the changes in speed during traffic adjustments are influenced by the time headway and reaction time. With a small time headway, vehicles can easily adjust to changes in traffic, ensuring string stability. This allows vehicles to maintain a consistent speed with few interruptions and quick driver response, reducing fuel consumption and emissions. Conversely, a large time headway requires larger vehicle adjustments, leading to less smooth traffic flow and increased stop-and-go traffic.

The string stability criteria for the ID model from (37) is.

39 $$\:{v}_{e}^{{\prime\:}}\left({h}_{e}\right)\le\:\frac{A\left(\begin{array}{c}\delta\:{h}_{e}^{2}{{v}_{e}\left({h}_{e}\right)}^{\delta\:-1}+2Tj{v}_{m}^{\delta\:}+\\\:2{v}_{e}{\left({h}_{e}\right)T}^{2}{v}_{m}^{\delta\:}\end{array}\right)}{2{\left({h}_{e}\right)}^{2}{v}_{m}^{\delta\:}}+\frac{{v}_{e}\left({h}_{e}\right)\sqrt{AB}\left({h}_{e}+T{v}_{e}\left({h}_{e}\right)\right)}{{\left({h}_{e}\right)}^{2}B}$$

This indicates that the changes in speed during traffic adjustments are influenced by the constant $\:\delta\:$. Traffic is more stable with a large value of $\:\delta\:$, resulting in quick dissipation of congestion. However, making the traffic stable with a larger value of $\:\delta\:$ is not based on traffic physics but rather is a parameter adjustment to ensure model and traffic stability. Thus, it results in an unrealistic and inadequate traffic characterization.

## Performance results

### Simulation setup and scenarios

The performance of the proposed model is compared with the ID model over a $\:1000$ m circular road for $\:800$ s with a platoon of 15 vehicles. The explicit Euler scheme with time step $\:0.5$ s is employed with both models to ensure accurate and stable results. The simulation parameters are given in Table 1. The desired speed is set to $\:{v}_{m}=30$ m/s^38^ and the maximum acceleration and deceleration are $\:1$ and $\:1.5$ m/s^2^, respectively^38^. The time headway for the ID model is $\:1.6$ s^35^ and the jam spacing is $\:7$ m^39^. The distance headway for the proposed model is $\:25$ m^44^, the safe distance headway is $\:5$ m^45^, and the jam spacing is $\:2$ m^38^. The proposed model is evaluated for $\:T=0.5,\:1.5$, and $\:2.5$ s as it is typically between $\:0.5-2.6$ s^46^. The ID model is evaluated for $\:\delta\:=1,\:12$, and $\:30$. With the ID model $\:\delta\:$ ranges from $\:1$ to $\:\infty\:$^35^ whereas with the proposed model its applicable range is between $\:\left|-0.003\right|$ and $\:\left|-0.0899\right|$ based on the realistic traffic parameters used from the literature. The CAV reaction time is $\:0.1$ s^46^.

**Table 1:** Simulation parameters.

Parameter	Value
Desired speed for the proposed and ID models, $\:{v}_{m}$	$\:30$ m/s
Time headway for the ID model, $\:T$	$\:1.6$ s
Time headway for the proposed model, $\:T$	$\:0.2,\:0.5,\:1.5,$ and $\:2.5$ s
Jam spacing for the ID model, $\:j$	$\:7$ m
Safe distance headway, $\:{h}_{s}$	$\:5$ m
Jam spacing for the proposed model, $\:j$	$\:2$ m
Maximum acceleration, $\:A$	$\:1$ m/s^2^
Distance headway, $\:h$	$\:25$ m
Deceleration, $\:B$	$\:1.5$ m/s^2^
Vehicle length,$\:\:L$	$\:4.5\:$m
Acceleration exponent, $\:\delta\:$	$\:1,\:12$, and $\:30$
Reaction time of CAV	$\:0.1$ s
Maximum normalized density for the proposed model, $\:{\rho\:}_{m}=1/j$	$\:0.5$
Maximum normalized density for the ID model, $\:{\rho\:}_{m}=1/j$	$\:0.14$
Time step, $\:\varDelta\:t$	$\:0.5$ s

### Traffic performance analysis and discussion

**Fig. 3:**
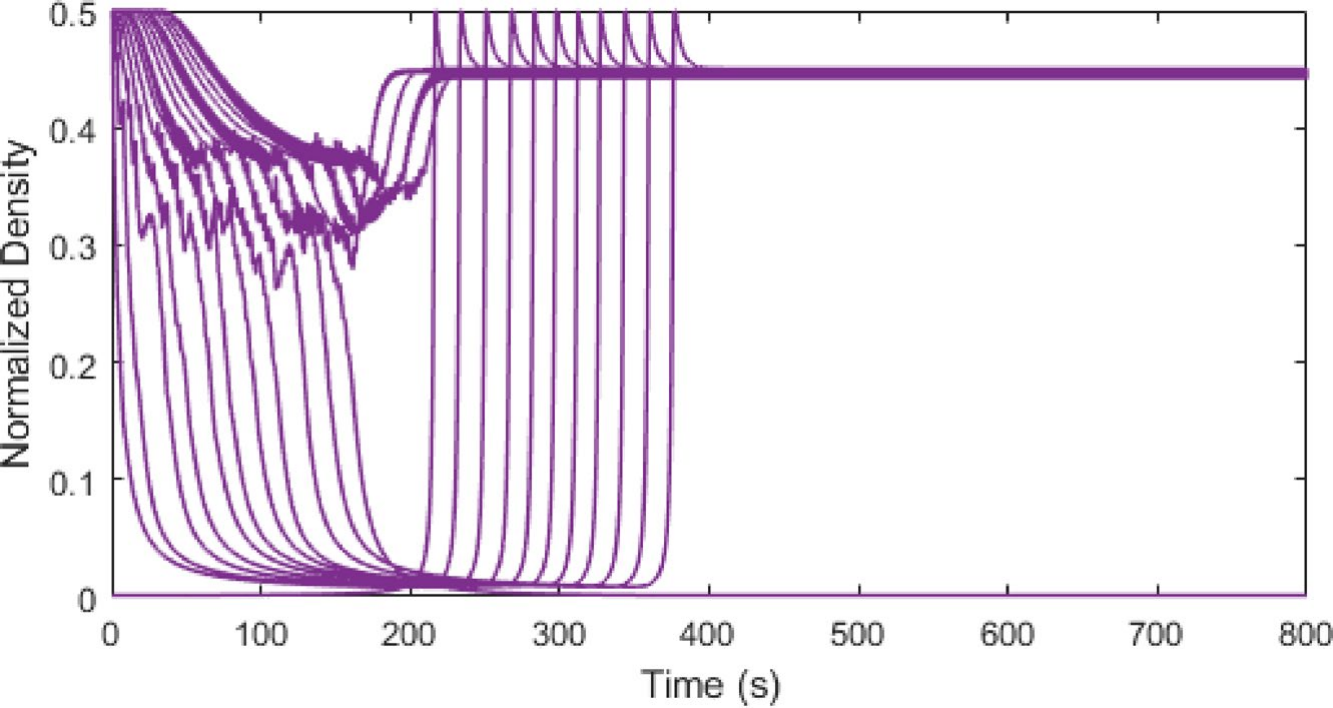
Traffic density evolution over time with the proposed model at $\:\mathrm{T}=0.2$ s on a $\:1000$ m road.

**Fig. 4:**
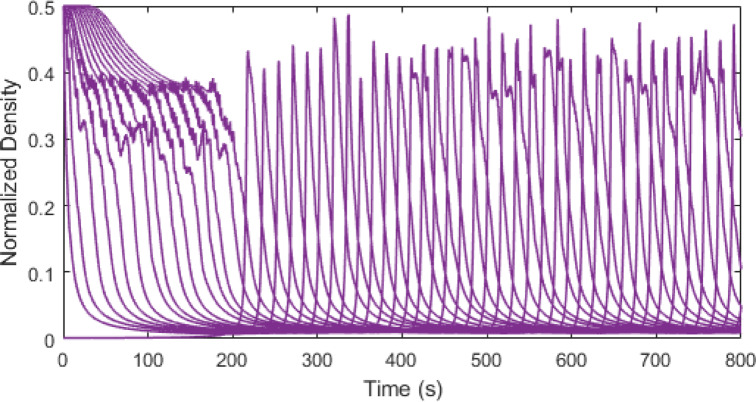
Traffic density evolution over time with the proposed model at $\:\mathrm{T}=0.5$ s on a $\:1000$ m road.

**Fig. 5:**
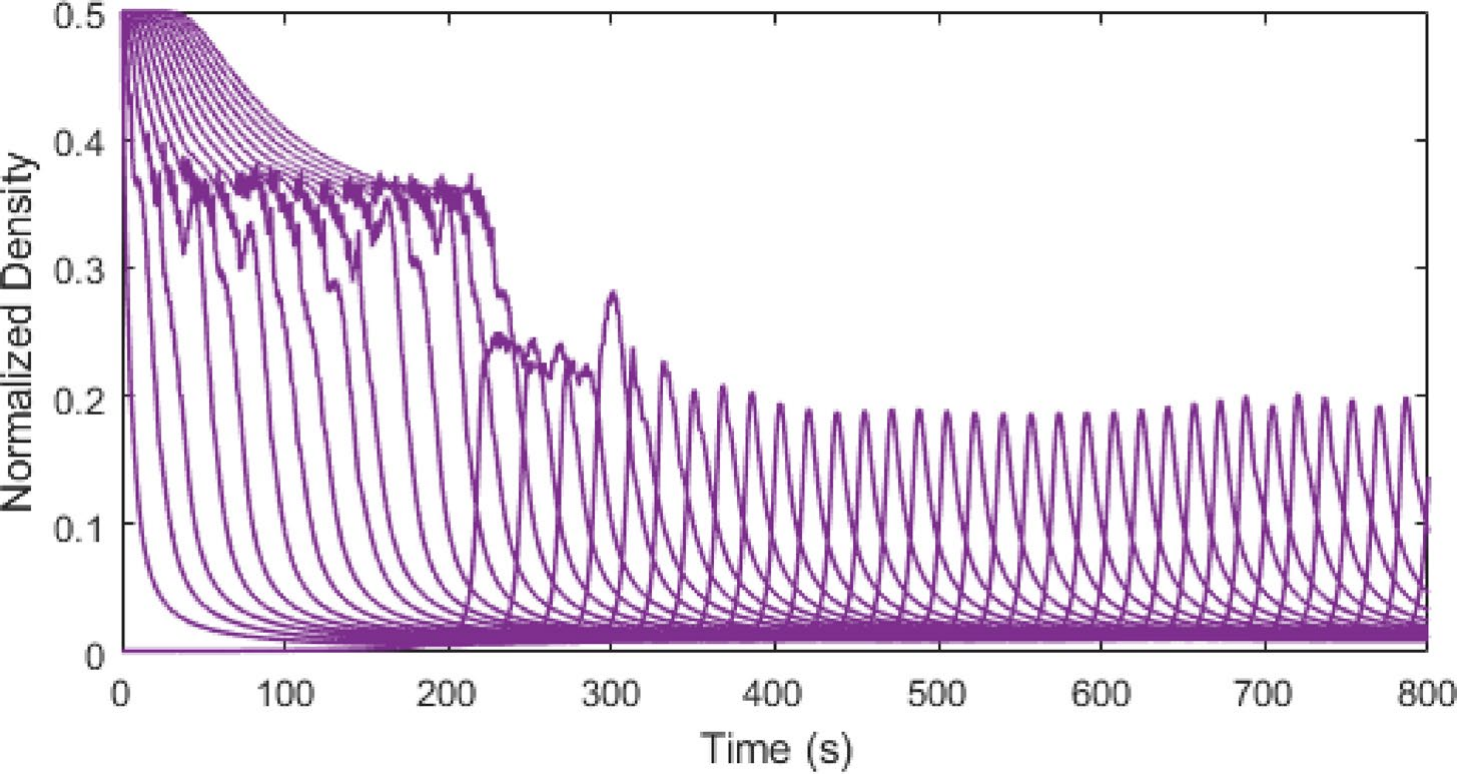
Traffic density evolution over time with the proposed model at $\:\mathrm{T}=1.5$ s on a $\:1000$ m road.

**Fig. 6:**
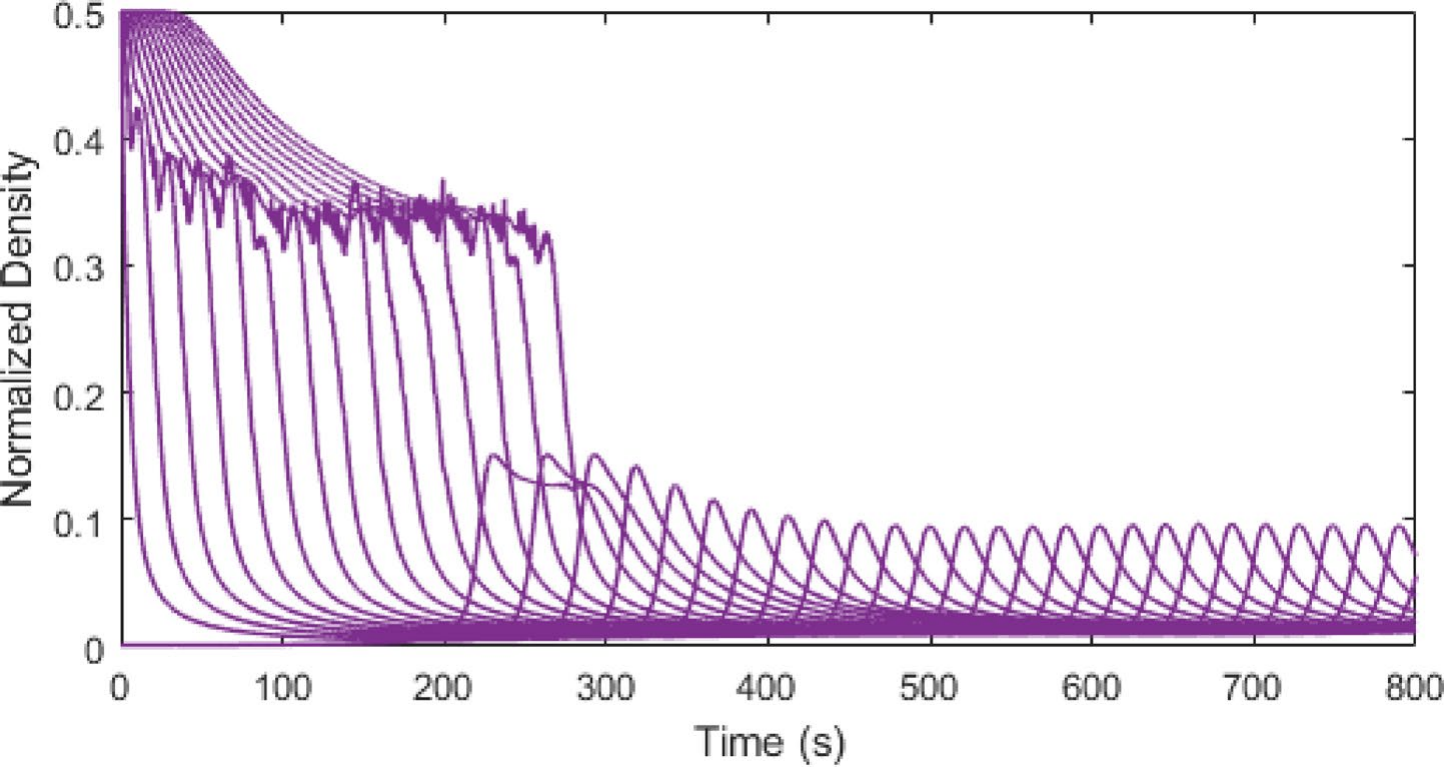
Traffic density evolution over time with the proposed model at $\:\mathrm{T}=2.5$ s on a $\:1000$ m road.

Figures 3, 4, 5 and 6 show traffic density evolution over time of all vehicles for the proposed model at time headway $\:\mathrm{T}=0.2,\:0.5,\:1.5$ and $\:2.5$ s on a $\:1000$ m road. This indicates the role of time headway in regulating vehicle interactions within the model. A longer time headway means the distance between vehicles is large, and vehicles will take more time to adapt to the forward traffic conditions. Thus, it leads to lower traffic density and smoother traffic flow. At a shorter time headway, $\:T=0.2$ s, the distance between vehicles is smallest, resulting in the highest density on the road with the initial density ranging between $\:0.01$ and $\:0.5$. The density then stabilizes at around $\:0.45$ and remains constant, indicating a congested traffic state. Likewise, at $\:\mathrm{T}=0.5$ s, the distance between vehicles is small and there are larger interactions between vehicles which leads to higher and more fluctuating density values, ranging between $\:0.01$ and $\:0.5$ as shown in Fig. 4. As the time headway increases, the distance between vehicles becomes large and at $\:\mathrm{T}=1.5$ s, the initial density remains similar ranging from $\:0.01$ to $\:0.5$ up to around $\:235$ s. Then the density stabilizes to a range of $\:0.01$ to $\:0.18$. This trend becomes more pronounced and at $\:\mathrm{T}=2.5$ s, the vehicles align more smoothly with a stronger reduction in density after $\:277$ s, ranging from $\:0.01$ to $\:0.09$. This shows that increasing time headway improves traffic stability by reducing density fluctuations. Consequently, lower density results in smoother traffic flow and lower emissions, highlighting the influence of time headway on traffic flow dynamics and emissions within the model.

**Fig. 7:**
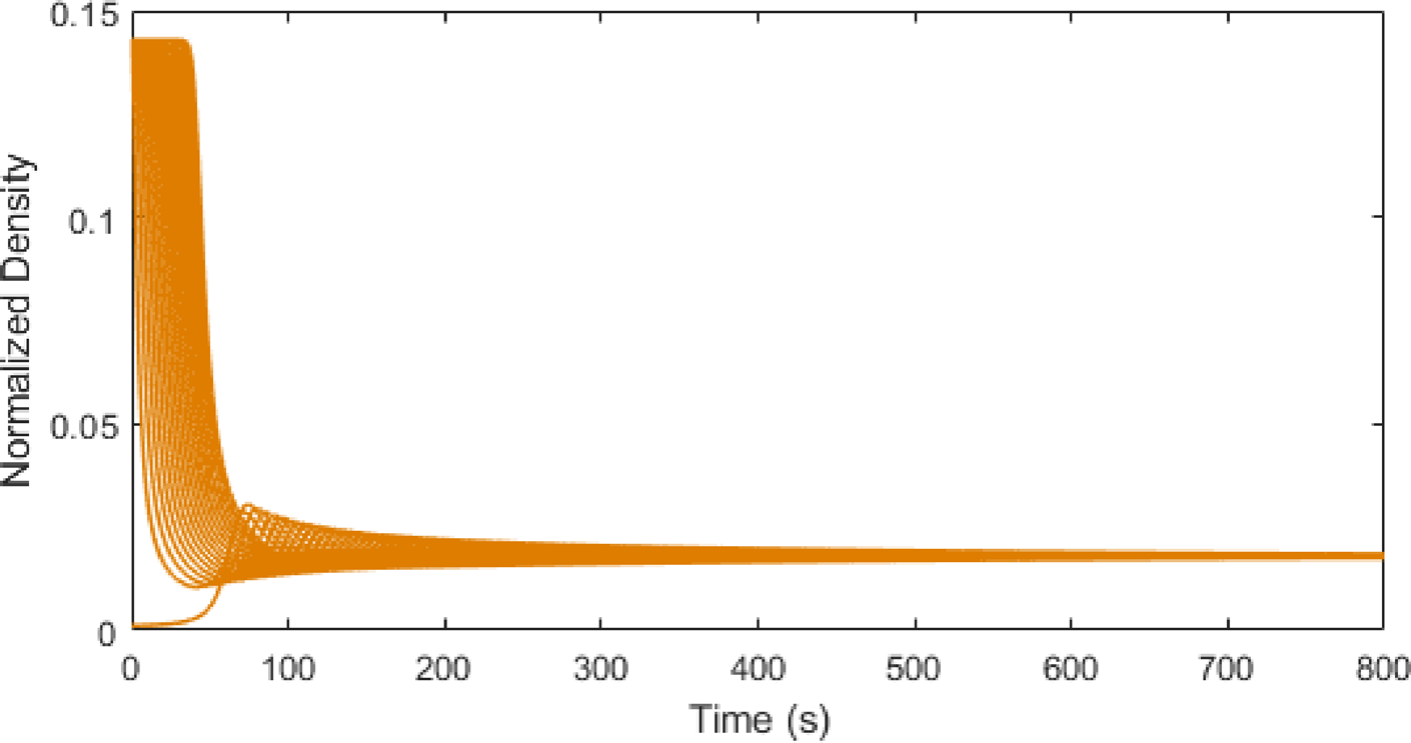
ID model traffic density evolution over time at $\:\delta\:=1$ on a $\:1000$ m road.

**Fig. 8:**
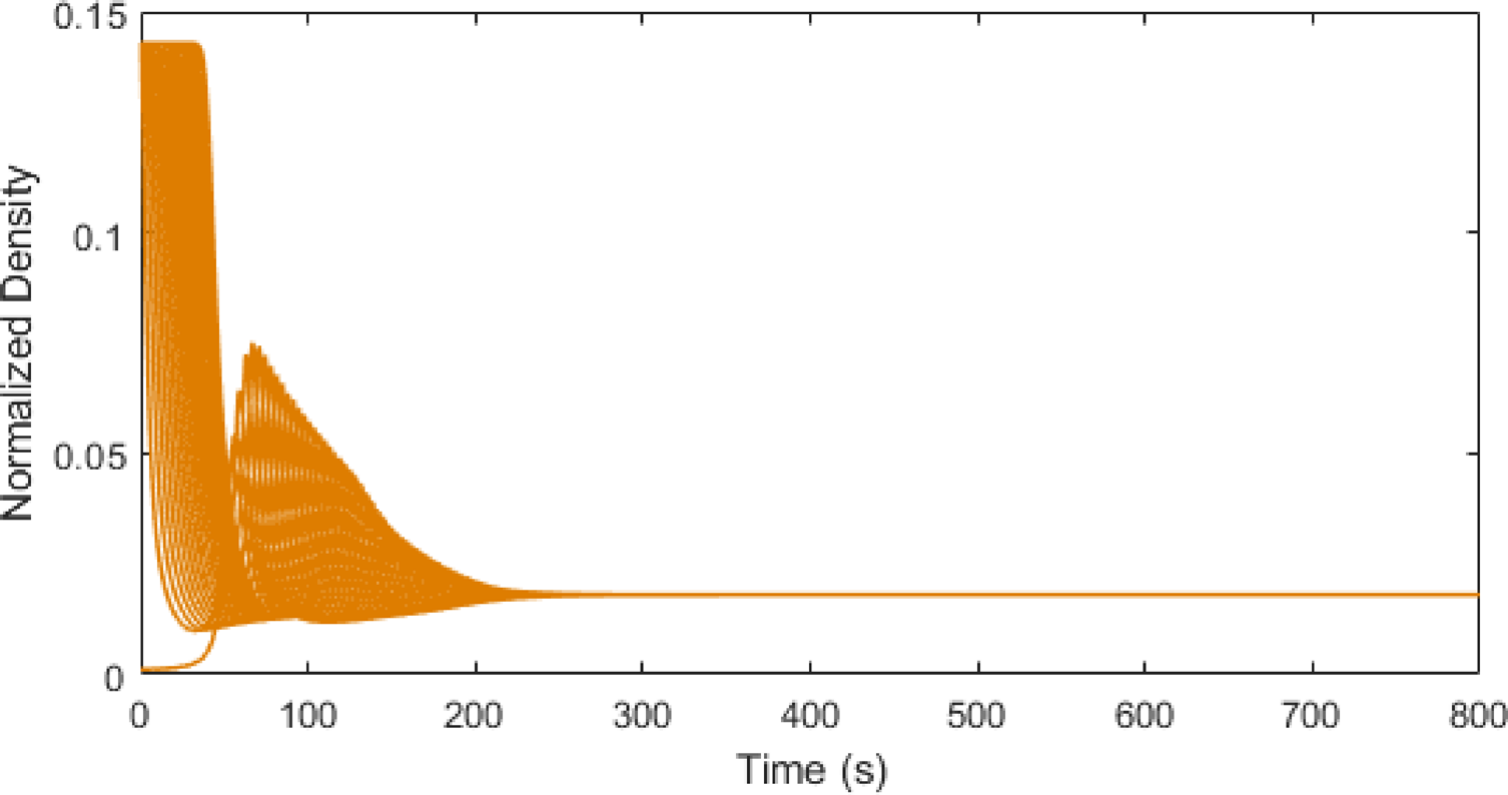
ID model traffic density evolution over time at $\:\delta\:=12$ on a $\:1000$ m road.

**Fig. 9:**
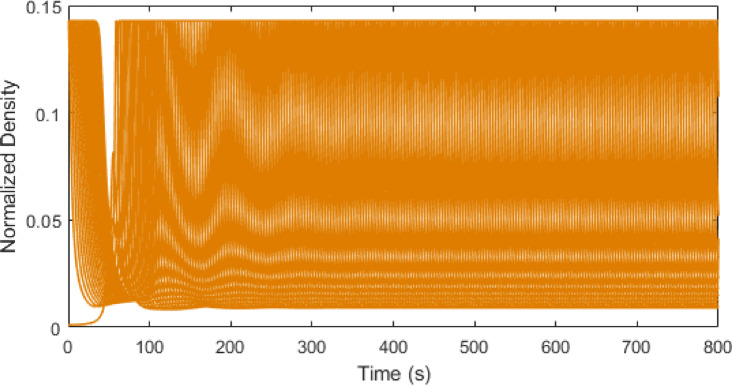
ID model traffic density evolution over time at $\:\delta\:=30$ on a $\:1000$ m road.

Figs. 7, 8 and 9 present the traffic density evolution of all vehicles with the ID model at $\:\delta\:=1,\:12$, and $\:30$. This illustrates that with a larger value of $\:\delta\:$ the traffic density increases, and the variations become larger. For $\:\delta\:=1$, the density is largely stable. After the initial variations between $\:0$ and $\:0.14$, the density reduces and remains low with small changes, indicating a smooth traffic flow. For $\:\delta\:=12$, the density initially varies between $\:0.01$ and $\:0.14$, and then the variations gradually decrease and the density becomes uniform. At $\:\delta\:=30$, the maximum traffic density is $\:0.14$ and remains high and variable, indicating congestion. However, this behavior is because of the model which is based on the constant $\:\delta\:$ which does not represent real-world traffic. The results show that increasing $\:\delta\:$ makes the model more sensitive, causing the fluctuations in density to grow and this has no physical meaning. Thus, the ID model does not capture real traffic behavior compared to the proposed model which is based on physical parameters such as time headway.

**Fig. 10:**
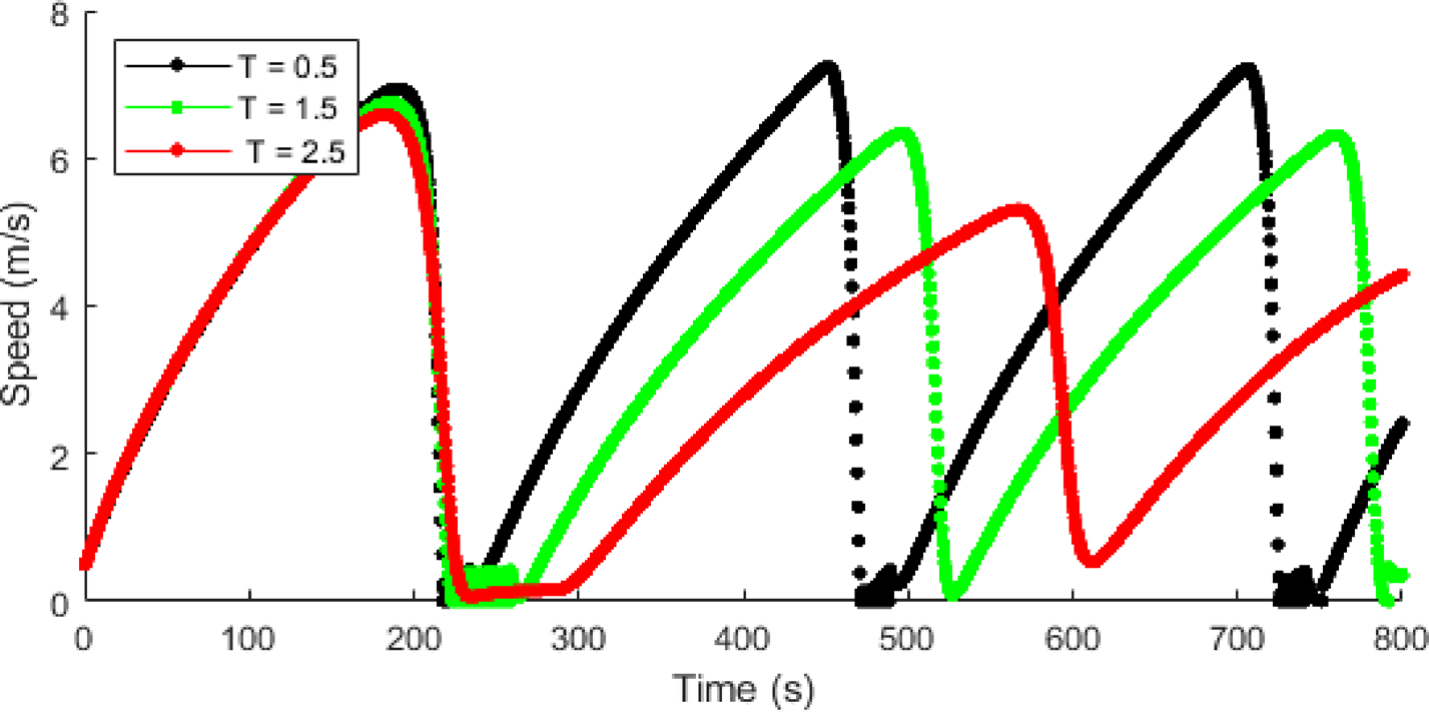
Single vehicle evolution of speed over time with the proposed model at $\:\mathrm{T}=0.5,\:1.5$, and $\:2.5$ s.

**Fig. 11:**
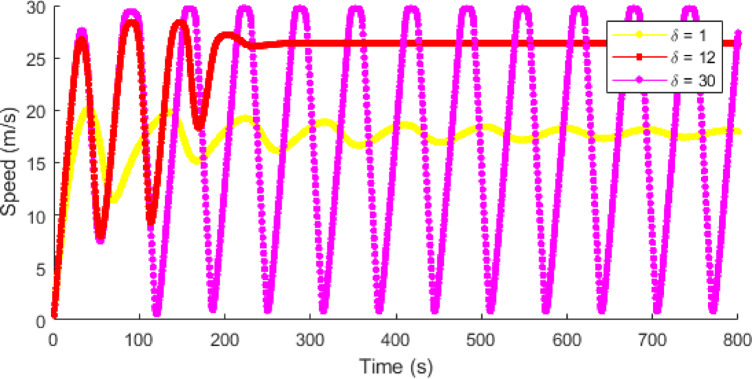
Single vehicle evolution of speed over time with the ID model at $\:\delta\:=1,\:12$, and $\:30$.

Figures 10 and 11 showsthat traffic speed evolution over time with the proposed model at $\:T=0.5,\:1.5$, and $\:2.5$ s, and ID model at $\:\delta\:=1,\:12$, and $\:30$, respectively. This shows that with the proposed model, as time headway increases vehicles have more time to adjust to forward traffic and hence the density is low, resulting in lower speed fluctuations and smoother traffic flow. At a short time headway of $\:T=0.5$ s, the interactions between vehicles are large due to higher density, leading to larger speed variations ranging from $\:0$ m/s to $\:7.2$ m/s. As time headway increases to $\:T=1.5$ s, there are small interactions between vehicles and the speed fluctuations decrease to a range of $\:0-6.7$ m/s. At $\:T=2.5$ s, the speed fluctuations further decrease, indicating improved traffic stability and smoother traffic as shown in Fig. 10. In contrast, ID model exhibits large speed fluctuations as the value of $\:\delta\:$ increases. At $\:\delta\:=1$, the model produces small variations in speed that gradually reduces over time. At $\:\delta\:=12$, there are large initial speed fluctuations ranging between $\:0.5$ m/s and $\:28.3$ m/s, which reduce over time and results in uniform speed of $\:26.3$ m/s. At $\:\delta\:=30$, the speed fluctuations remain large and persistent ranging between $\:0.5$ m/s and $\:29.6$ m/s, indicating an instable traffic behavior. However, the fluctuations produced in speed are due to a constant $\:\delta\:$ rather than physical vehicle interactions and results in unrealistic traffic behavior.

**Fig. 12:**
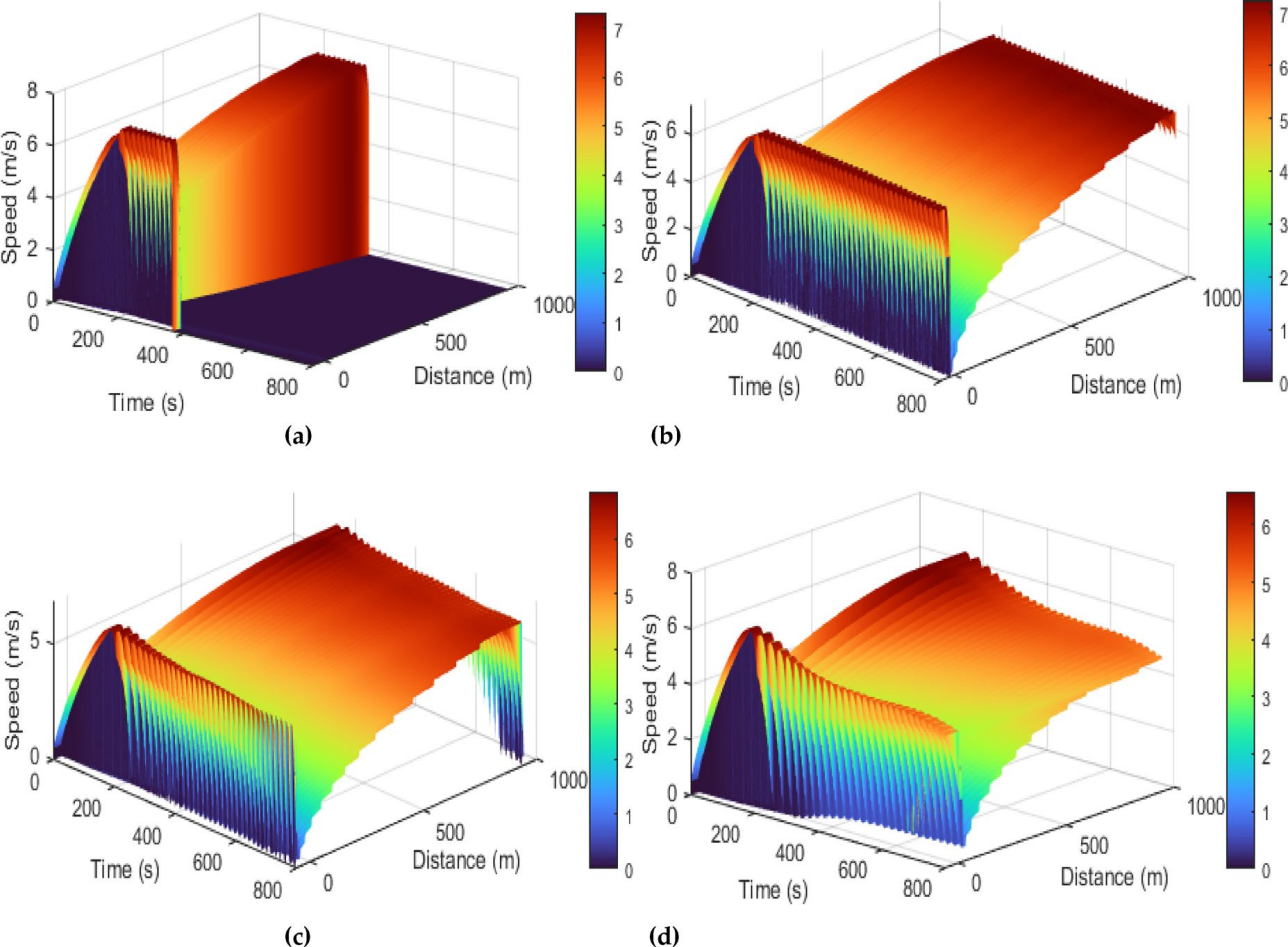
Traffic speed behavior with the proposed model at (**a**) $\:T=0.2$ s, (**b**) $\:T=0.5$ s, (**c**) $\:T=1.5$ s, and (**d**) $\:T=2.5$ s.

**Fig. 13:**
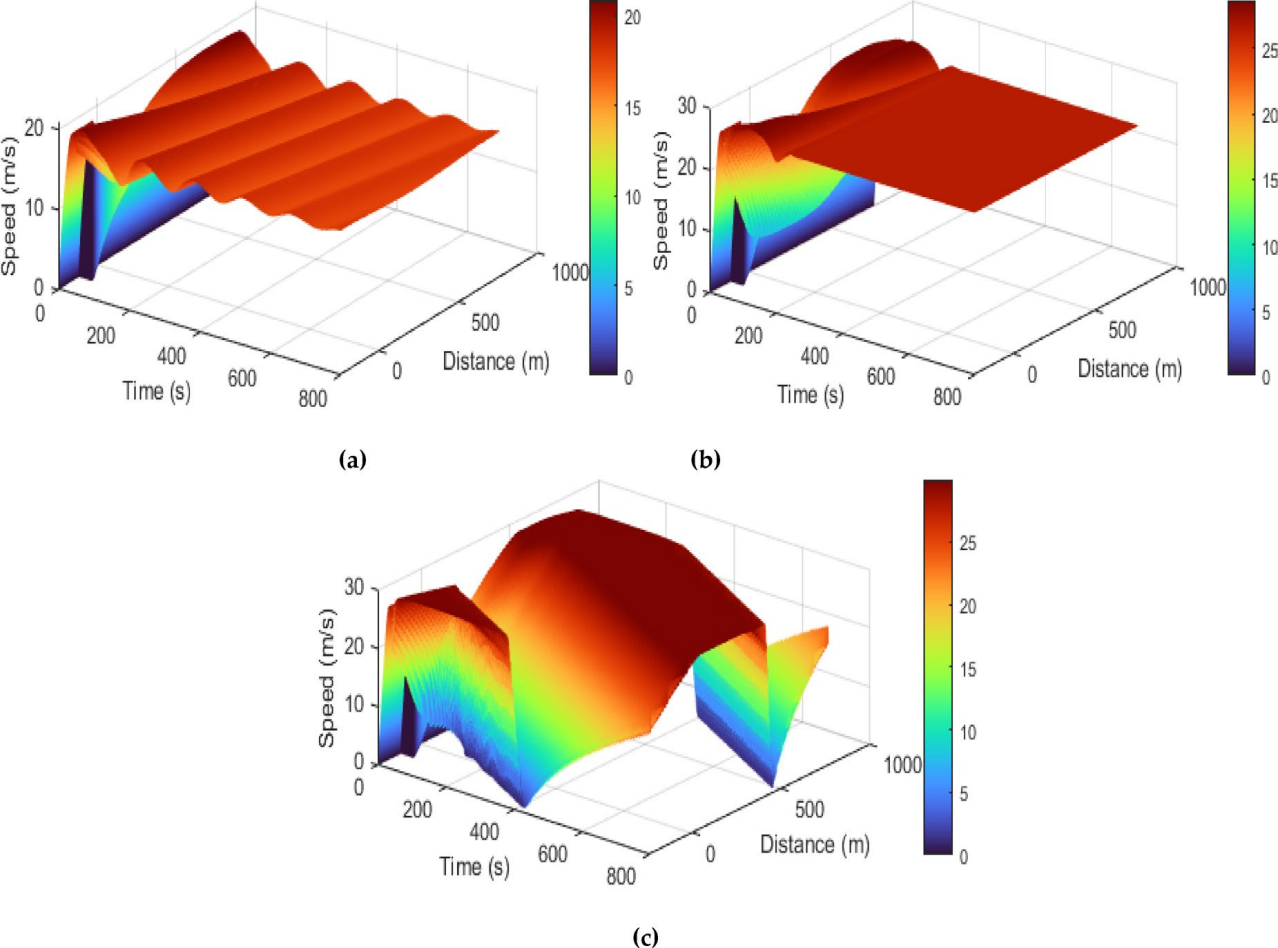
Traffic speed behavior with the ID model at (**a**) $\:\delta\:=1$, (**b**) $\:\delta\:=12$, and (**c**) $\:\delta\:=30$.

Figures 12 and 13 present the speed behavior over time and space with the proposed and ID models, respectively. With the proposed model, a small time headway of $\:T=0.2$ s represents highly dense traffic conditions and therefore results in congestion, with the speed dropping to $\:0$ m/s after the initial variations. As the time headway increases, the proposed model yields smooth and gradually varying speed over time and space. Further, lower traffic speeds are observed with the proposed model because CAVs regulate their motion to maintain safe distances between vehicles and minimize acceleration. This reduces stop-and-go traffic behavior and the associated emissions. Conversely, the ID model results in higher speeds and pronounced fluctuations that increase with $\:\delta\:$. The large variations in speed result in frequent acceleration and deceleration, and thus high traffic emissions.

**Fig. 14:**
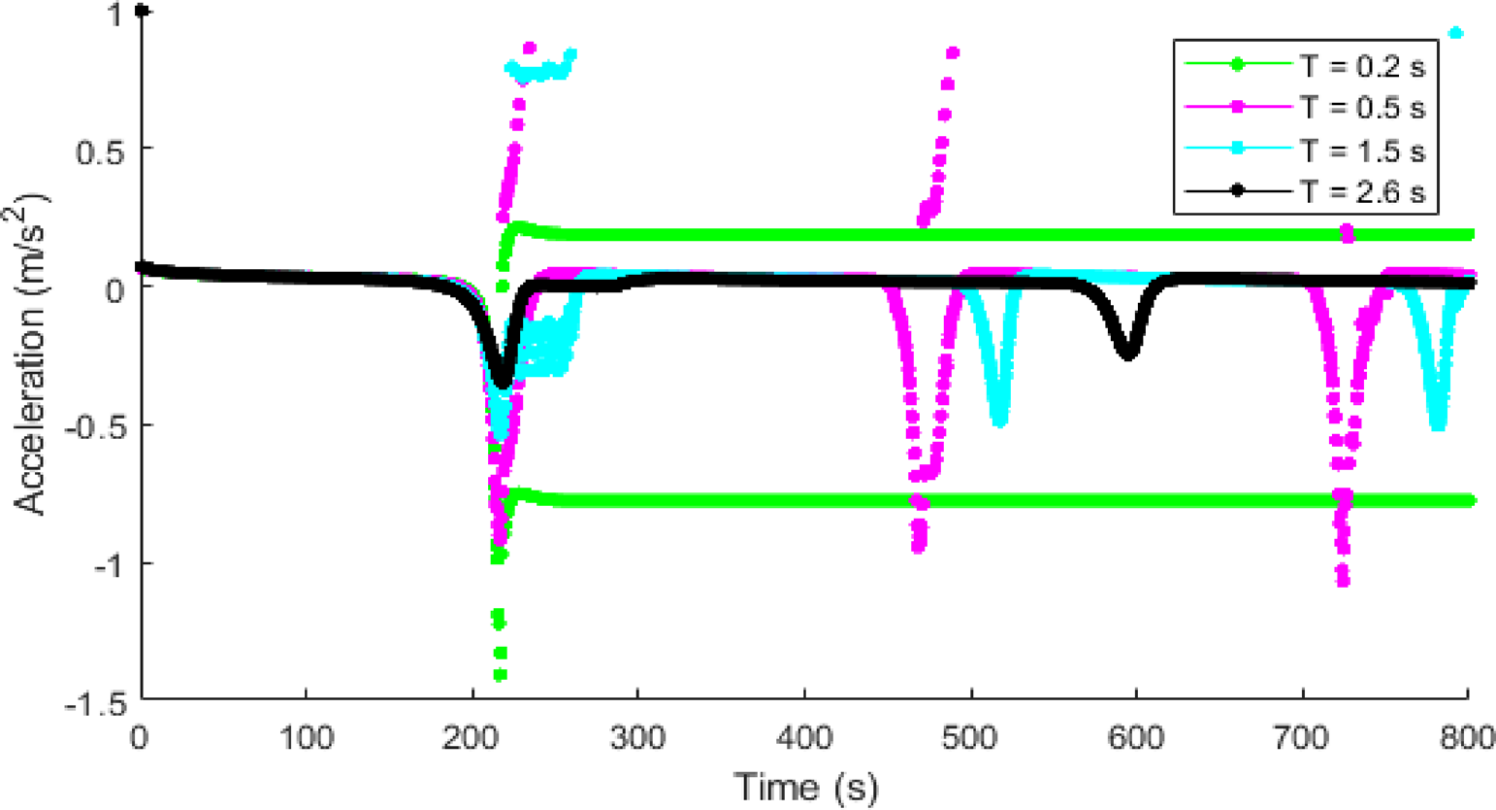
Acceleration evolution of a single vehicle with the proposed model at $\:\mathrm{T}=0.2,\:0.5,\:1.5$ and $\:2.5$ s.

**Fig. 15:**
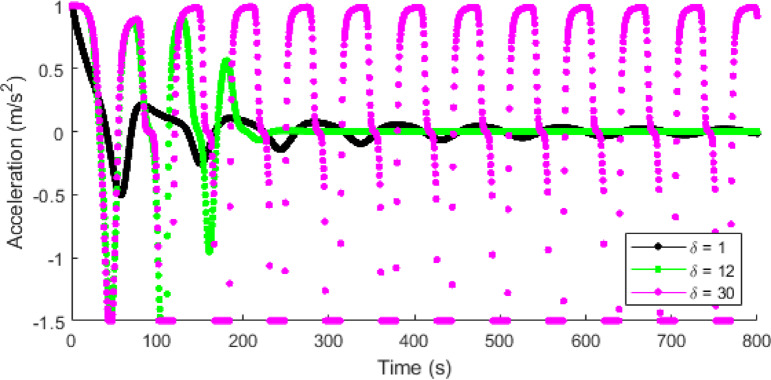
Acceleration evolution of a single vehicle with the ID model at $\:\delta\:=1,\:12$, and $\:30$.

Figures 14 and 15 present the temporal acceleration with the proposed model at $\:T=0.2,\:0.5,\:1.5$, and $\:2.5$ s, and the ID model at $\:\delta\:=1,\:12$, and $\:30$, respectively. The proposed model with a shorter time headway of $\:T=0.2$ s indicates a highly dense traffic condition with a consistent variation in acceleration and deceleration between $\:0.18$ m/s^2^ and $\:-0.77$ m/s^2^ as shown in Fig. 14. The variations in acceleration decrease with an increase in $\:T.$ Consequently, a longer time headway of $\:T=2.5$ s corresponds to a lower traffic density and results in lower acceleration with negligible variations. This leads to smoother traffic behavior and reduced emissions. Conversely, the ID model results in large acceleration and deceleration variations that become pronounced with increased $\:\delta\:$. At $\:\delta\:=1$, the acceleration fluctuations are moderate and gradually decay over time, but at $\:\delta\:=30$, the variations are large and persist between $\:1$ m/s^2^ and $\:-1.5$ m/s^2^, thereby resulting in large traffic emissions.

**Fig. 16:**
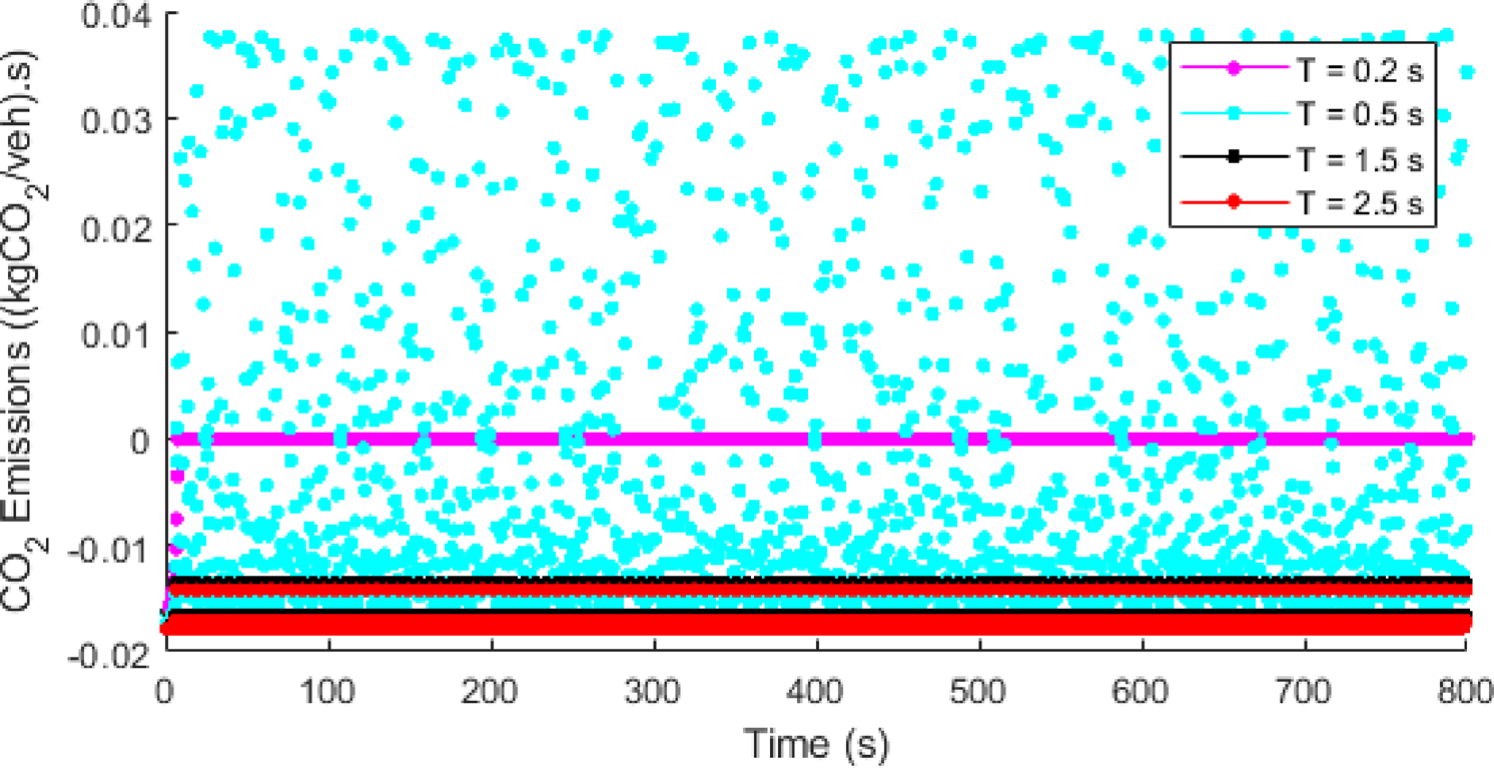
Temporal evolution of the sensitivity of $\:\mathrm{C}{\mathrm{O}}_{2}$ emissions to traffic density with the proposed model at $\:\mathrm{T}=0.2,\:0.5,\:1.5$, and $\:2.5$ s.

Figure 16 presents the temporal evolution of $\:\mathrm{C}{\mathrm{O}}_{2}$ emissions with respect to traffic density for the proposed model at $\:\mathrm{T}=0.2,\:0.5,\:1.5$, and $\:2.5$ s, highlighting the sensitivity of emissions to traffic changes. At a shorter time headway of $\:T=0.2$ s, both the magnitude and fluctuations of $\:\mathrm{C}{\mathrm{O}}_{2}$ emissions remain relatively low. This is because with a short time headway traffic is congested and driving behavior is constrained, resulting in comparatively lower and stable emission levels. As the time headway increases to $\:T=0.5$ s, the emission sensitivity increases, leading to highly fluctuating emission levels and unstable traffic conditions. At a longer time headway of $\:T=1.5$ s and $\:T=2.5$ s, the increased distance between vehicles reduces traffic density, leading to lower and more stable emissions.

Overall, the results show that with the proposed model an increase in time headway improves traffic stability by reducing fluctuations in speed, density, and acceleration, as a large distance between vehicles allows more time to adjust to forward traffic and the density is low. Furthermore, a longer time headway leads to lower and more stable $\:\mathrm{C}{\mathrm{O}}_{2}$ emissions. In contrast, with the ID model a large value of $\:\delta\:$ results in higher fluctuations in speed, density, and acceleration, leading to an unstable traffic flow and higher emissions. The behavior of the ID model is based on the constant $\:\delta\:$ which does not have any physical meaning. Therefore, while the ID model can generate different traffic patterns, it cannot capture real-world traffic behavior. Conversely, the proposed model is based on physical traffic parameters and so provides a more realistic and emission-efficient representation of traffic dynamics.

### Quantitative analysis

The speed, density, and acceleration statistics for the proposed model and ID model are presented in Tables 2 and 3, respectively. With the proposed model at $\:T=0.2$ s, the mean speed is low, that is, $\:1.25$ m/s, and the median speed is almost zero, indicating frequent stop-and-go behavior. The standard deviation of speed is $\:2.22$ m/s and the acceleration variability is $\:0.44$ m/s². These relatively high values reflect an unstable traffic condition. Further, a high mean density of $\:0.33$ and substantial variability of $\:0.19$, which indicates congestion. At $\:T=0.5$ s, the mean and median speed increase to $\:3.73$ m/s and $\:3.99$ m/s, respectively, indicating an improved traffic stability. Further, the mean density reduces, reflecting a reduction in congestion. At $\:T=1.5$ s, the mean speed decreases to $\:3.57$ m/s, and both mean density and density standard deviation are further reduced, indicating smoother traffic. At $\:T=2.5$ s, the mean density is lowest, indicating a free flow of traffic. Overall, the results show that increasing time headway leads to a reduction in standard deviation of speed, density, and acceleration, indicating an improved traffic stability. The acceleration standard deviation decreases from 0.44 m/s² to 0.063 m/s², corresponding to an $\:86$% reduction in acceleration variability, which indicates lower emissions.

Conversely, the ID model exhibits an increase in standard deviation of speed, density, and acceleration as $\:\delta\:$ increases. At $\:\delta\:=1$, the mean speed is high with a standard deviation of $\:2.11$ m/s, and the average density is low, indicating a stable motion. At $\:\delta\:=12$, the standard deviation of speed and acceleration increase to $\:4.44$ m/s and $\:0.36$ m/s², respectively, and the acceleration median is $\:0$ m/s², reflecting oscillatory behavior. At $\:\delta\:=30$, the model results in highly unstable traffic. The standard deviation of speed and density increases sharply to $\:9.68$ m/s and $\:0.05$, while the standard deviation of acceleration increases to $\:1.00$ m/s², corresponding to a six times increase in variability than $\:\delta\:=1$. This increase in variability indicates that the model increasingly amplifies disturbances, leading to stronger acceleration–deceleration cycles, and thereby resulting in large emissions.

**Table 2:** Speed, density, and acceleration with the proposed model.

Time headway	Parameter	Mean	Median	Standard deviation	Coefficient of variation
$\:T=0.2$ s	Speed	1.25	0.09	2.22	1.77
	Density	0.33	0.45	0.19	0.59
	Acceleration	-0.21	0.03	0.44	-2.06
$\:T=0.5$ s	Speed	3.73	3.99	2.34	0.63
	Density	0.04	0.01	0.08	1.98
	Acceleration	-0.002	0.03	0.17	-73.80
$\:T=1.5$ s	Speed	3.57	3.86	2.09	0.58
	Density	0.03	0.01	0.05	1.68
	Acceleration	-0.00038	0.02	0.133	-350.20
$\:T=2.5$ s	Speed	2.97	3.04	1.89	0.63
	Density	0.02	0.01	0.03	1.31
	Acceleration	0.005	0.018	0.06	-11.45

**Table 3:** Speed, density, and acceleration with the ID model.

Acceleration Exponent	Parameter	Mean	Median	Standard Deviation	Coefficient of variation
$\:\boldsymbol{\delta\:}=1$	Speed	17.30	17.72	2.11	0.12
	Density	0.01	0.01	0.004	0.26
	Acceleration	0.02	0.01	0.15	6.93
$\:\boldsymbol{\delta\:}=12$	Speed	24.86	26.39	4.44	0.17
	Density	0.01	0.02	0.006	0.34
	Acceleration	0.03	0.00	0.36	11.15
$\:\boldsymbol{\delta\:}=30$	Speed	17.32	17.88	9.68	0.55
	Density	0.05	0.02	0.05	1.10
	Acceleration	$\:0.03$	$\:0.35$	$\:1.00$	$\:29.32$

**Table 4:** Statistics of $\:\mathrm{C}{\mathrm{O}}_{2}$ emissions sensitivity ((kg$\:\mathrm{C}{\mathrm{O}}_{2}$/veh).s) with the proposed model.

Time headway	Min	Max	Mean	Median	Standard deviation	Coefficient of variation
$\:T=0.2$ s	− 0.016	0.000	− 0.0001	0.000	0.0013	$\:-10.691$
$\:T=0.5$ s	− 0.017	0.038	− 0.001	-0.008	0.016	$\:-13.500$
$\:T=1.5$ s	− 0.018	− 0.013	− 0.017	-0.017	0.0011	$\:-0.067$
$\:T=2.5$ s	− 0.019	− 0.014	− 0.017	-0.018	0.0001	$\:-0.057$

Table 4 presents a statistical summary of $\:\mathrm{C}{\mathrm{O}}_{2}$ emissions sensitivity to traffic density ((kg$\:\mathrm{C}{\mathrm{O}}_{2}$/veh).s) with the proposed model at $\:T=0.2,\:0.5,\:1.5$, and $\:2.5$ s. At a shorter time headway of $\:T=0.2$ s, $\:\mathrm{C}{\mathrm{O}}_{2}$ emissions exhibit a small mean value of approximately zero and a relatively large standard deviation. This results in large variability and a coefficient of variation of $\:-10.691$, indicating an unstable emission behavior. The variability is even more pronounced at $\:T=0.5$ s, where emissions exhibit a wider range between minimum and maximum values and the highest coefficient of variation of $\:-13.500$, resulting in very unstable emission behavior among all the scenarios. Conversely, longer time headways of $\:T=1.5$ s and $\:T=2.5$ s exhibit more stable emission characteristics, with a closely clustered minimum and maximum values and low coefficient of variation of $\:-0.067$ and $\:-0.057$, respectively. This indicates low variability, signifying that a large distance between vehicles (low traffic density) leads to smoother driving behavior and more consistent $\:\mathrm{C}{\mathrm{O}}_{2}$Overall, the analysis presented in this section supports simulation results and indicates that the proposed model explicitly accounts for emissions and produces more stable, realistic, and emission efficient traffic behavior compared to the ID model.

## Conclusion

In this paper, a new vehicle $\:\mathrm{C}{\mathrm{O}}_{2}$ emissions model based on the traffic density was developed. The relation between density and $\:\mathrm{C}{\mathrm{O}}_{2}$ emission was obtained through the regression technique by utilizing the data acquired from the roadside. CAV behavior is incorporated, and a new microscopic model that includes traffic emissions considering CAV behavior was proposed. In this model, driving behavior governs traffic dynamics and emissions are predicted as an outcome. The intelligent driver (ID) model employs a constant exponent, so traffic behavior is the same regardless of the conditions, and emissions and CAV behavior are not considered. The traffic speed, density, and acceleration predicted by the proposed model could provide the details of vehicle emissions under varying traffic densities. The proposed and ID models were compared over a $\:1000$ m circular road in MATLAB. The results show that with the proposed model the variations in traffic speed, density, and acceleration based on the traffic emissions are small and realistic compared to the ID model. Furthermore, they demonstrate that the proposed model with a longer headway leads to lower and more consistent $\:\mathrm{C}{\mathrm{O}}_{2}$ emissions, highlighting the role of density in influencing traffic related environmental impacts.

The changes in speed, density, and acceleration with the ID model are based on the constant $\:\delta\:$, thus resulting in inadequate characterization. Conversely, the proposed is based on real-world data from the roadside and therefore more accurately characterizes traffic behavior. In addition, the stability analysis showed that traffic with the proposed model is more stable. Moreover, the variability with the ID model is higher when $\:\delta\:$ is higher, indicating unstable and inefficient traffic behavior. This can result in significantly higher traffic emissions compared to the proposed model.

The proposed model can be utilized by traffic planners and operators to identify traffic conditions where emissions are likely to be high and evaluate control strategies such as coordinated speeds, eco-driving measures, and CAV-based traffic control. Further, it can be employed to design congestion mitigation strategies and mitigate the adverse impact of $\:\mathrm{C}{\mathrm{O}}_{2}$ emissions on air quality and climate change. Future research can consider incorporating factors such as vehicle type, road geometry, and weather conditions to provide results for a wider range of scenarios.

## Data Availability

All the data generated is included in the manuscript.
